# Influence of surgical margins on overall survival after resection of intrahepatic cholangiocarcinoma: A meta-analysis

**DOI:** 10.1097/MD.0000000000004621

**Published:** 2016-09-02

**Authors:** Haowen Tang, Wenping Lu, Bingmin Li, Xuan Meng, Jiahong Dong

**Affiliations:** a Hospital and Institute of Hepatobiliary Surgery, Chinese PLA General Hospital; b Medical School of Chinese PLA, Haidian; c Center for Hepatopancreatobiliary Diseases, Beijing Tsinghua Changgung Hospital, Tsinghua University Medical Center, Changping, Beijing, China.

**Keywords:** Intrahepatic cholangiocarcinoma, meta-analysis, negative, overall survival, surgical margin length

## Abstract

**Background:**

Surgical resection is shown to present the best chance of cure in the treatment of intrahepatic cholangiocarcinoma (ICC). However, the appropriate length of the negative margin remains unclear. The aim of the present meta-analysis was to investigate whether a clear margin of 10 mm or more (≥10 mm) conferred any survival benefit over a margin of less than 10 mm (<10 mm) in patients with resected ICC.

**Methods:**

The meta-analysis was conducted in adherence with the PRISMA guidelines. PubMed, Web of Science, EMBASE, and the Cochrane Library were systematically searched to identify eligible studies published in English from the initiation of the databases to February 2016. Overall survival rates were pooled by using the hazard ratio and the corresponding 95% confidence interval (CI). Random-effect models were utilized because of between-study heterogeneity.

**Results:**

Six studies (eight cohorts) reporting on 712 patients were analyzed: 269 (37.80%) were in the 10 mm or more negative margin group, and 443 (62.20%) were in the less than 10 mm negative margin group. The pooled hazard ratio for the less than 10 mm group was found to be 1.59 (95% CI: 1.09–2.32) when this group was compared with the 10 mm or more group (reference), with moderate between-study heterogeneity (*I*^2^ = 45.30%, *P* = 0.07). Commensurate results were identified by sensitivity analysis.

**Conclusion:**

The result of this meta-analysis suggests a long-term survival (overall survival) advantage for negative margins of 10 mm or more in comparison with negative margins less than 10 mm for patients undergoing surgical resection of ICC.

## 1 Introduction

Intrahepatic cholangiocarcinoma (ICC) is the second most common primary hepatic malignancy arising from the epithelial lining of the intrahepatic bile duct beyond the second-order bile ducts.^[1,2]^ Based on its anatomical location, ICC constitutes one of the three categories of cholangiocarcinoma (intrahepatic, hilar, and extrahepatic). As a poorly understood variant of cholangiocarcinoma, ICC has been considered to be the least common category among the above 3 from a histological point of view. The last 3 decades have witnessed a global increase in the incidence of and mortality from ICC, while the incidence of all other forms of cholangiocarcinoma has been stable or has declined.^[3–6]^ The rarity of ICC poses a great obstacle not only to understanding of the disease's pathogenesis but also to the development of effective treatment approaches.

Surgery remains the first-line approach and presents the best chance of cure in the treatment of ICC. However, the surgical result is not fully satisfactory, with a 5-year survival rate ranging from only 30% to 35%,^[7]^ which is significantly lower than after resection of hepatocellular carcinoma (HCC)^[8]^ or colorectal liver metastases (CRLM).^[9]^ Perhaps more worryingly, little improvement in this rate has been achieved over the past decade^[1,10]^ in comparison with the rate for HCC^[11]^ or CRLM.^[12]^ Moreover, several clinicopathological factors influencing overall survival (OS) after resection of HCC or CRLM have been well delineated, such as margin adequacy. For CRLM, it was recently emphasized that margin status, rather than margin length, determined long-term survival.^[9,13,14]^ Conversely, for HCC, patients with wider negative margins were demonstrated to have improved long-term outcomes.^[15,16]^ However, controversy still exists regarding the appropriate length of surgical margins in ICC resection. Previous literature^[17]^ argued that the length of a clear resection margin had no impact on long-term survival, whereas other reports^[18,19]^ documented long-term survival benefits in patients with clear margins of 10 mm or more in length compared with those with margins of less than 10 mm. In the present study, a 10-mm negative margin was chosen as the cutoff for the following reasons. With regard to the impact of margin length on outcome in the resection of ICC, most surgeons strive to achieve a negative margin of 10 mm.^[20]^ Additionally, a 10-mm negative margin strikes a balance between surgical curability and functional preservation of the remnant liver. Hence, a meta-analysis was conducted to investigate whether a clear margin of 10 mm or more (≥10 mm) conferred any survival benefit over a margin of less than 10 mm (<10 mm) for patients with resected ICC.

## 2 Methods

The meta-analysis was conducted in adherence with the recommendations of the Preferred Reporting Items for Systematic Reviews and Meta-Analyses (PRISMA) guidelines.^[21,22]^ All analyses were based on previously published studies, thus no ethical approval and patient consent are required. To ensure accuracy and minimize bias, all vital stages of the analysis were carried out separately by 2 reviewers; any disagreement was settled through consensus discussion.

### 2.1 Study selection

PubMed, Web of Science, EMBASE, and the Cochrane Library were systematically searched to find relevant articles published in English from the initiation of the databases to February 2016. No extra restrictions were applied to the search strategies. Search terms include both relevant medical subject headings (MeSH) and keywords. The following MeSH were used: “Bile Ducts, Intrahepatic,” “Bile Duct Neoplasms,” “Cholangiocarcinoma,” “Biliary Tract Surgical Procedures,” and “Hepatectomy.” And the keywords below were also used to complete the literature searches: “Cholangiocarcinoma,” “Cholangiocellular Carcinoma,” “Peripheral,” “Intrahepatic,” “Hepatectomy,” “Liver resection,” and “Margin.” In addition, the reference lists of retrieved articles were manually screened for potentially relevant studies. For dual or multiple studies describing the same population, either the most recent or the highest in quality was selected. For inclusion in our analysis, studies (cohorts) had to meet the following 4 inclusion criteria: ICC (confirmed by pathological examination) patients primarily undergoing potentially curative resections; inclusion of surgical margins as a variable in the outcome analysis; stratification of negative surgical margins into less than 10 mm (with or without additional subgroups) and 10 mm or more groups; and a survival hazard ratio (HR) for a less than 10 mm group compared with a 10 mm or more group, either directly available in the article or possible to calculate. The exclusion criteria were as follows: articles with the types of abstracts, reviews, case reports, editorials, and expert opinions; articles grouping the patients by other cutoff values of margin length; overlapping or duplicate reports; articles including patients mainly undergoing repeated hepatectomy for recurrent ICC; and articles including patients with extrahepatic metastases (metastases in the lung, bone, or brain). A study (cohort) meeting any of the 5 exclusion criteria was excluded.

### 2.2 Data extraction and definition

Two investigators (HT and BL) extracted and summarized the following pertinent parameters from the included studies (cohorts): first author's name, publication year, study type (retrospective or prospective), origin of study (population), recruitment period, number of patients with negative margins (R0 resection), numbers of patients enrolled in less than 10 mm group and 10 mm or more group, substratifications of the less than 10 mm group with their corresponding numbers of enrolled patients, liver parenchymal dissection techniques, proportion of lymph node involvement and dissection, tumor size and subtype, maximum follow-up length, and HR for OS. Outcomes from multivariate analyses were superior to those from univariate analyses for inclusion if both were presented. For studies substratifying the less than 10 mm group into different substratifications,^[20,23]^ corresponding substratification HRs (reference ≥10 mm) were separately taken into account using the method described by Botteri E.^[24]^ One study^[25]^ ultimately included categorized negative margins in a “10 mm or less and more than 10 mm” group. For the purpose of unified grouping and pooled analysis, 10 mm or less was treated as less than 10 mm, and more than 10 mm was treated as 10 mm or more. In the absence of primary patient data, this method would surely result in misclassification of patients with exactly 10 mm margins as patients with less than 10 mm margins and thus relatively bias the result toward a positive outcome for less than 10 mm margins. However, a seemingly more conservative approach was used, as defined by an earlier report.^[26]^ If the HR, the 95% confidence interval (CI), or additional key data were absent from an article, the corresponding author of the report was contacted by e-mail. In the absence of a reply from the author, the methods introduced by Tierney were used to derive an approximate estimation of the HR and corresponding CI from other information, such as the Kaplan–Meier curve for OS.^[27]^

In the present study, only patients with negative margins (R0 resection) were eligible to be included. Liver dissection techniques mainly denoted the techniques or devices used in the dissection of the liver parenchyma. In our study, lymphatic invasion and lymphadenectomy mostly referred to lymph node involvement and lymph node dissection, respectively, at the site of the hepatoduodenal ligament (regional lymph nodes, N1 disease based on the 7th edition of the American Joint Committee on Cancer [AJCC] staging system). According to the macroscopic appearances proposed by the Liver Cancer Study Group of Japan, ICC includes 3 subtypes: the mass-forming (MF) subtype, the periductal infiltrating subtype, and the intraductal growth subtype. Subtypes were defined based on preoperative imaging and the macroscopic description in the pathological report. A quality assessment of each included article was conducted using the Newcastle–Ottawa Scale, which is mainly concerned with three aspects (selection of patients, comparability of groups, and assessment of outcomes). Studies scored with 6 or more were considered to be of high quality. Subgroups were generated if at least 2 studies (or 2 cohorts) were available; otherwise, subgroup analyses were not performed. A 2-tailed *P* value less than 0.05 was considered statistically significant.

### 2.3 Outcome comparison and statistical analysis

For comparison of OS, the HR with a 95% CI was used. An HR value (reference ≥10 mm) greater than one indicated a survival benefit favoring the 10 mm or more group over the less than 10 mm group. For comparison of categorical variables, the χ^2^ test or Fisher's exact test was utilized, as appropriate. A random-effect model was used for the existence of moderate between-study heterogeneity. Meta-analysis was performed using STATA statistical software (version 12.0, Stata Corporation, College Station, TX). Cochrane's *Q* and *I*^2^ tests were utilized to test the between-study heterogeneity. Publication bias was assessed by Begg funnel plot and Egger tests. Sensitivity analysis was conducted by omitting the included studies sequentially. Subgroup analyses were performed according to the following four predefined parameters: cohorts with all MF subtype, cohorts without lymph node involvement and cohort sample size (size ≥50 or size <50). Meta-regression analysis was not conducted due to the limited number of studies included; this analysis is best suited to analyzing at least 10 studies.

## 3 Results

### 3.1 Study selection and patient characteristics

**Figure 1:**
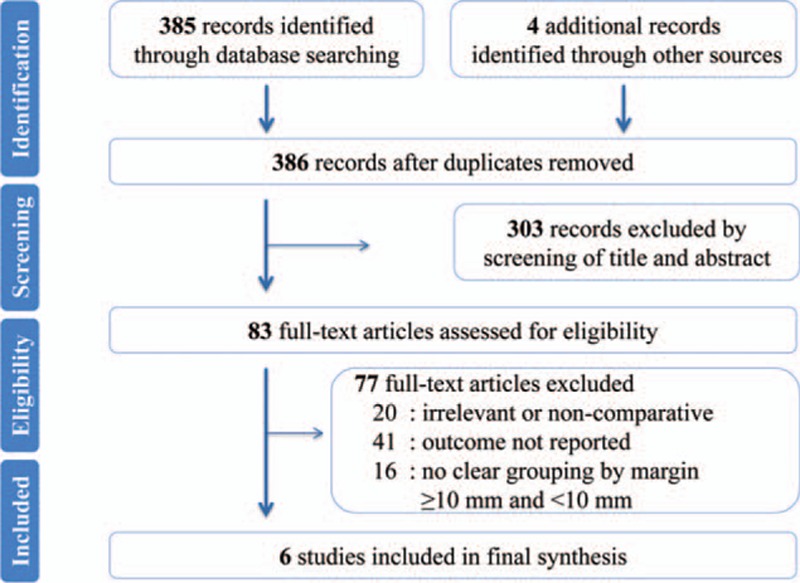
PRISMA flowchart of the study selection.

Figure 1 illustrates the PRISMA flowchart of study selection process. By removal of duplicates, a total of 386 references was obtained using the aforementioned search strategy. After rigorously skimming the titles and abstracts, 303 references were judged irrelevant to our topic and eliminated. The remaining 83 studies were further checked by full-text analysis, and 77 were excluded as follows: 20 were irrelevant or noncomparative studies; 41 studies lacked outcomes of interest; 16 studies lacked clear groupings by margin (<10 mm and ≥10 mm). Ultimately, 6 studies (8 cohorts) involving 712 patients were included in the final synthesis.^[18–20,23,25,28]^ All 6 studies were retrospective, nonrandomized studies published between 1995 and 2015 that were conducted in the Republic of Korea (1 study), Japan (1 study), Austria (1 study), or France (1 study) or that were multicenter (2 studies). Among the 712 patients enrolled, 269 (37.80%) were in the 10 mm or more group, and 443 (62.20%) were in the less than 10 mm group. The median sample size for these studies (cohorts) was 63.5 (range 14–340). For most studies (cohorts), the mean or median age of the patients was in the 60s or 70s, and the median male patient percentage was 57.29% (range 40.00%–63.50%; the aforementioned results are not shown in Table 1). For most of the patients enrolled, hepatic resection was carried out mainly using a variety of modern techniques or dissection devices, such as the Cavitron ultrasonic surgical aspirator or a waterjet or ultrasonic dissector. Based on the available data from 5 studies (7 cohorts), the tumor size and the proportions of solitary lesions and MF subtypes were similar between the 2 groups (≥10 mm margin group and <10 mm margin group). The maximum follow-up length in the studies ranged from 36 to 115 months. The study (cohort) characteristics and quality assessment are summarized in Table 1, and the main outcomes are outlined in Table 2.

**Table 1:**
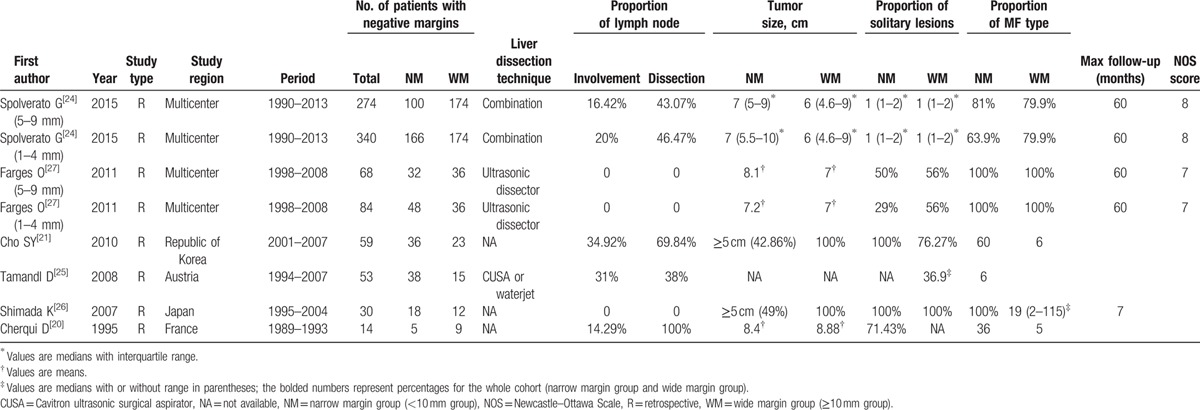
Characteristics of included studies (cohorts).

**Table 2:**
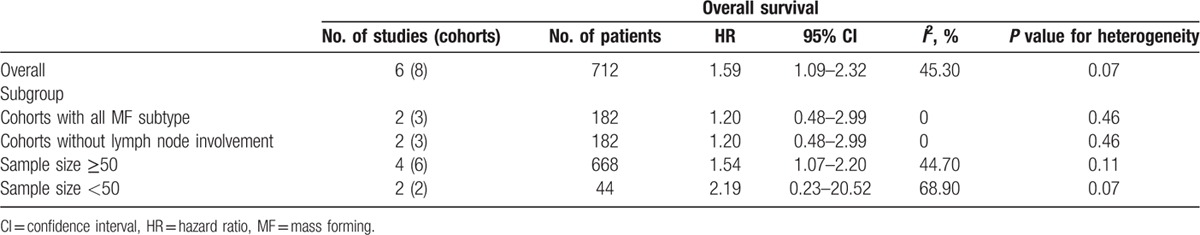
Overall and subgroup results of the meta-analysis.

### 3.2 Survival hazard ratios

**Figure 2:**
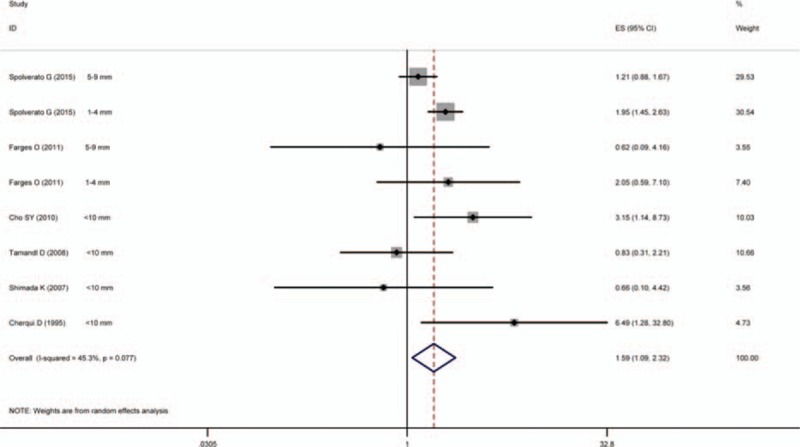
Results of the meta-analysis of pooled HR values. In the case of studies substratifying the less than 10 mm group into different substratifications (1–4 and 5–9 mm), corresponding substratification HRs (reference ≥10 mm) were used separately. CI = confidence interval, HR = hazard ratio.

The pooled HR for the less than 10 mm negative margin group was found to be 1.59 (95% CI: 1.09–2.32) when compared with the HR for the 10 mm or more group (reference), with moderate between-study heterogeneity (*I*^2^ = 45.30%, *P* = 0.07). A statistically significant survival benefit was identified in patients with negative margins 10 mm or more relative to those with negative margins less than 10 mm. Figure 2 illustrates the results of the pooled analysis.

### 3.3 Subgroup analyses

In accordance with 4 predefined parameters, namely, cohorts with all MF subtype, cohorts without lymph node involvement and cohort sample size (size ≥50 or size <50), subgroup analyses were conducted. Pooled analyses showed similar result in comparison with the overall findings, except that no significant differences were identified between the 10 mm or more margin group and the less than 10 mm margin group in the following 3 subgroups: cohorts with all the MF subtype (HR 1.20, 95% CI: 0.48–2.99), cohorts without lymph node involvement (HR 1.20, 95% CI: 0.48–2.99), and cohorts with a sample size less than 50 (HR 2.19, 95% CI: 0.23–20.52). All the aforementioned results are detailed in Table 2.

### 3.4 Analysis of sensitivity and test for publication bias

**Figure 3:**
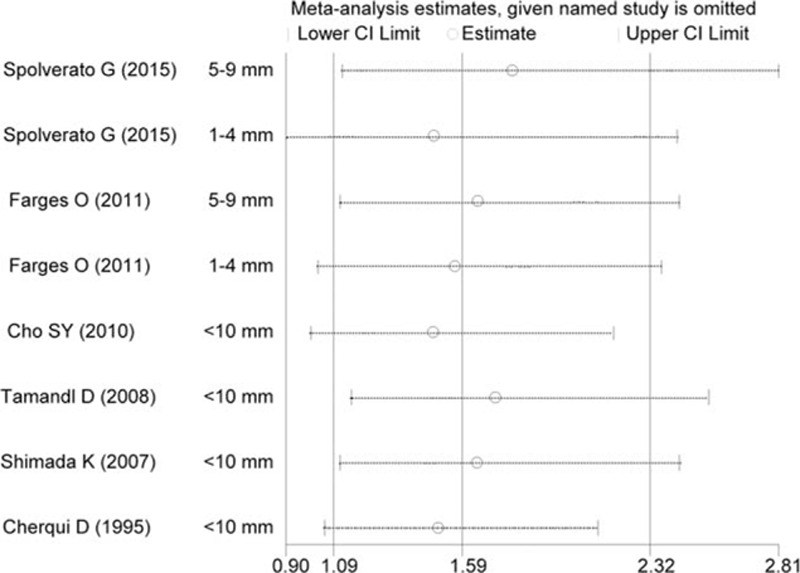
Results of the sensitivity analysis. The middle vertical line indicates the combined HR, and the 2 vertical lines represent the corresponding 95% CI values. The middle small circle and the 2 ends of the dotted lines indicate the pooled HR and 95% CI values, respectively, when the study on the left was omitted after each round of analysis. CI = confidence interval, HR = hazard ratio.

**Figure 4:**
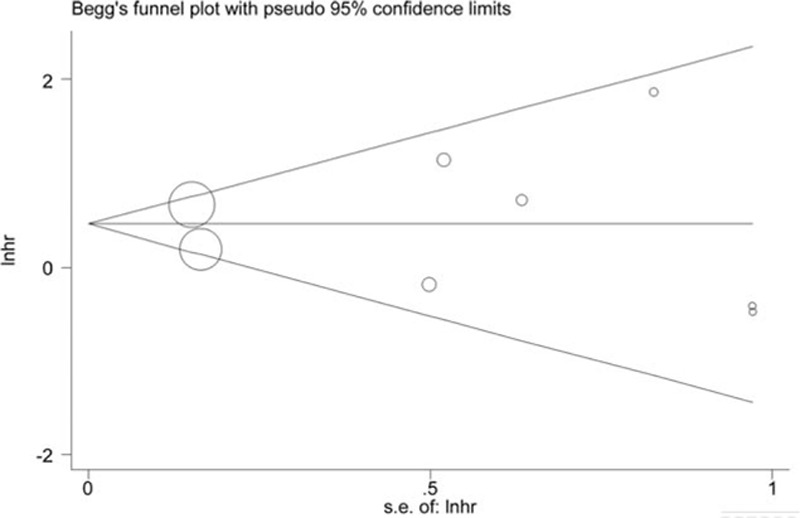
Begg's funnel plot to evaluate OS. CI = overall survival.

The result of sensitivity analysis demonstrated no significant changes in HR values (range: 1.01–1.66, Fig. 3). Similarly, no obvious publication bias was detected by Egger test (*P* = 0.99), with symmetry in Begg funnel plot (Fig. 4). However, it must be admitted that the relatively limited studies for inclusion might influence the statistic power of the aforementioned results.

## 4 Discussion

Despite consensus that a negative margin portends a better outcome in comparison with a positive margin, the most appropriate length of the negative margin after resection of ICC remains controversial. In fact, to the best of our knowledge, this is the first meta-analysis addressing the impact of negative margin length on survival outcomes for patients with ICC. The present study has reviewed the long-term survival differences between patients with negative margins of 10 mm or more and those with negative margins less than 10 mm after resection of ICC. The results showed that patients with negative margins of 10 mm or more enjoyed a survival advantage when compared with patients with negative margins less than 10 mm (HR 1.59, 95% CI: 1.09–2.32). A commensurate result was identified by sensitivity analysis.

Intuitively reasonable as the results are, the specific reasons for a survival benefit among patients with 10 mm or more negative margins are beyond the purview of the present study. However, the findings might be explained by the particular disseminating mode of ICC. It was previously reported that ICC was likely to have a particular disseminating mode involving direct sinusoidal invasion into the adjacent liver parenchyma, without a tumor capsule, vascular invasion, and perineural invasion, causing micrometastases or tumor cells rooting in the surrounding liver parenchyma.^[29]^ Most of the metastatic deposits were primarily confined to the 10 mm region surrounding the primary lesion border. Consequently, a large proportion of such circumferential or sideward deposits would be eradicated by a resection margin of more than 10 mm. This finding was consistent with previous literature arguing OS as incrementally worsening as the margin length decreased from 10 mm. OS HRs for patients with negative margins of 1 to 4 mm and 5 to 9 mm were 1.95 (reference ≥10 mm, 95% CI: 1.45–2.63) and 1.21 (reference ≥10 mm, 95% CI: 0.88–1.68), respectively.^[20]^ A consistent result was again obtained by Cherqui D.^[18]^ In his report, patients meeting 3 criteria (no lymph node invasion, a negative margin of more than 10 mm, and the presence of only a solitary tumor) enjoyed a survival rate of 100% in both 1- and 2-year periods. Furthermore, previous literature documented that a negative margin of 1 mm or less was equivalent to a positive margin, with similar median survival lengths (15 and 12 months, respectively) and no 3-year survivors. In comparison, patients with a margin wider than 10 mm had a median survival of 64 months and a 5-year survival rate of 68%.^[23]^ These findings were confirmed by both univariate and multivariate analyses in reported series revealing that a resection margin less than 10 mm independently predicted a dismal prognosis.^[19]^

Subgroup estimations in the present meta-analysis demonstrated a prognostic significance of negative margins of 10 mm or more for a long-term survival (OS) advantage in ICC patients for some of the subgroups. However, no such significant differences between less than 10 mm and 10 mm or more groups were found in the following subgroups: cohorts with all the MF subtype, cohorts without lymph node involvement, and cohorts with a sample size of less than 50. Such inconsistent results might be in part due to the limited studies for inclusion (only 2 studies for each subgroup) and the obvious between-study heterogeneity (eg, *I*^2^ = 68.90% in the subgroup with a sample size <50).

Of note, apart from the negative margin length, other clinicopathological features, such as lymph node involvement, lymph node dissection, the number of tumors (single or multiple), and tumor size, have been identified by the majority of studies as the most important determinants of prognosis in patients with ICC after operation.^[7,10,25,30–32]^ In particular, multiple studies have identified the presence of lymph node involvement as the most important independent prognostic factor in ICC.^[7,30–32]^ However, due to the strict patient selection criteria in the present meta-analysis, most features remained commensurate between groups. For instance, the results of the χ^2^ test for lymph node involvement (*P* = 0.07), the proportion of solitary lesions (*P* = 0.07), and the MF type (*P* = 0.08) revealed that the differences among the included groups were of no statistical significance. Meanwhile, modern techniques or devices were used in liver transection for most of the patients (Table 1); these techniques may widen patient-specific resection margins and render assessment of the margins more accurate. Taken together, the results of the present study serve as a relatively persuasive argument for margin adequacy in ICC.

However, it must be admitted that none of the studies included in our meta-analysis discussed the impact of the type of liver resection (anatomical resection or nonanatomical resection according to the 2000 International Hepato-Pancreato-Biliary Association Brisbane Terminology of Liver Anatomy and Resections) on the outcome of ICC. To date, few studies have investigated the issue of whether anatomical resection presents prognostic advantages over nonanatomical resection for the treatment of ICC. The prognostic effect of the type of resection on the outcome thus remains inconclusive and warrants further scrutiny.

Our present study has 3 main strengths: to date, to the best of our knowledge, this is the first meta-analysis addressing margin adequacy in patients with resected ICC. Using relatively strict study selection criteria (inclusion and exclusion) and categorization of the margin length, a total of 712 patients were included, forming a substantial retrospective cohort from which to make clinically reasonable assumptions about patients with this uncommon pathology. In sensitivity and subgroup analyses, similar results were obtained, thus confirming the overall findings. Hence, our results were reliable and robust.

Despite the aforementioned improvements, certain limitations of the present study should be taken into consideration. The main limitation is that the size of the studies included, particularly in the subgroup analyses, was rather small, given the rarity of ICC. Moreover, no randomized controlled trials were available for inclusion, which reduced the reliability of the results. In addition, the lack of relevant data did not permit more subgroup analysis according to additional parameters, such as liver parenchymal dissection techniques, tumor size, and tumor differentiation (despite the fact that most of the parameters remained comparable between studies, as shown in Table 1), to be conducted. Finally, certain HR values were calculated using corresponding Kaplan–Meier curves for OS because of the unavailability of these values in the articles and the absence of replies from the authors. Nevertheless, the present study undoubtedly represents one more step in developing a persuasive argument for margin adequacy in ICC.

## 5 Conclusion

In summary, the result of this meta-analysis suggests a survival advantage for negative margins of 10 mm or more in comparison with negative margins less than 10 mm for patients undergoing surgical resection of ICC. However, because such a wide surgical margin may not be feasible in every case, a resection margin less than 10 mm should not be recognized as a contraindication to surgery. Taken together, the findings suggest that surgeons ought to strive to achieve a negative margin of 10 mm or more in surgical resection of ICC to obtain a long-term survival (OS) benefit. Further multicenter and high-quality randomized controlled trials will be required to support this conclusion.
